# Multiscale entropy analysis of biological signals: a fundamental bi-scaling law

**DOI:** 10.3389/fncom.2015.00064

**Published:** 2015-06-02

**Authors:** Jianbo Gao, Jing Hu, Feiyan Liu, Yinhe Cao

**Affiliations:** ^1^Institute of Complexity Science and Big Data Technology, Guangxi UniversityNanning, China; ^2^PMB Intelligence LLCSunnyvale, CA, USA; ^3^School of Management, University of Chinese Academy of SciencesBeijing, China

**Keywords:** scaling law, multiscale entropy analysis, fractal signal, heart rate variability (HRV), adaptive filtering

## Abstract

Since introduced in early 2000, multiscale entropy (MSE) has found many applications in biosignal analysis, and been extended to multivariate MSE. So far, however, no analytic results for MSE or multivariate MSE have been reported. This has severely limited our basic understanding of MSE. For example, it has not been studied whether MSE estimated using default parameter values and short data set is meaningful or not. Nor is it known whether MSE has any relation with other complexity measures, such as the Hurst parameter, which characterizes the correlation structure of the data. To overcome this limitation, and more importantly, to guide more fruitful applications of MSE in various areas of life sciences, we derive a fundamental bi-scaling law for fractal time series, one for the scale in phase space, the other for the block size used for smoothing. We illustrate the usefulness of the approach by examining two types of physiological data. One is heart rate variability (HRV) data, for the purpose of distinguishing healthy subjects from patients with congestive heart failure, a life-threatening condition. The other is electroencephalogram (EEG) data, for the purpose of distinguishing epileptic seizure EEG from normal healthy EEG.

## 1. Introduction

Biological systems provide the definitive examples of highly integrated systems functioning at multiple time scales. Neurons function on a time scale of milliseconds. Circadian rhythms operate on time scale of hours, reproductive cycles occur on a time scale of weeks, and bone remodeling involves time scales of months. As an integrated system, each process interacts with faster and slower processes. Consequently, biosignals often are multiscaled (Gao et al., 2007)—depending upon the scale at which the signals are examined, they may exhibit different behaviors (e.g., nonlinearity, sensitive dependence on small disturbances, long memory, extreme variations, and nonstationarity), just as a great painting may exhibit various details and arouse a multitude of aesthetic feelings when appreciated at different distances, from different angles, under different illuminations, and under different moods.

With the rapid advance of sensing technology, complex data have been accumulating exponentially in all areas of life sciences. To better cope with such complex data, recently, Costa et al. (2005) have introduced an interesting method, the multiscale entropy (MSE) analysis. MSE has found numerous applications in various types of biosignal analysis, including fetal heart rate monitoring (Cao et al., 2006), assessment of EEG dynamical complexity in Alzheimer's disease (Mizuno et al., 2010), classification of surface EMG of neuromuscular disorders (Istenic et al., 2010), heart rate analysis for predicting hospital mortality (Norris et al., 2008), and analysis of hear beat interval and blood flow for characterizing psychological dimensions in non-pathological subjects (Nardelli et al., 2015). MSE has also been extended to multivariate MSE (Ahmed and Mandic, 2011) and multiscale permutation entropy (Li et al., 2010). So far, however, no analytic analyses about MSE or multivariate MSE have been carried out. This has severely limited our basic understanding of MSE. For example, it has not been known whether MSE estimated using default parameter values and short data set is meaningful or not. Nor is it known whether MSE has any relation with other complexity measures, such as the Hurst parameter, which characterizes the correlation structure of the data.

To help gain insights into the above questions, and to guide more fruitful applications of MSE in diverse fields of life sciences, in this work, we report a fundamental bi-scaling law for MSE of the most popular model of biosignals, the fractal 1/*f* type time series. As example applications, we will analyze heart rate variability (HRV) and electroencephalogram (EEG) data. With HRV, we will focus on distinguishing healthy subjects from patients with congestive heart failure (CHF), a life-threatening condition, as well as resolving an interesting debate (Wessel et al., 2003; Nikulin and Brismar, 2004) regarding the usefulness of MSE in distinguishing HRV of healthy subjects from that of patients with certain cardiac disease. With EEG, we will focus on distinguishing epileptic seizure EEG from normal healthy EEG.

## 2. Materials and methods

### 2.1. Data

To illustrate the use of scaling analysis of MSE, in this paper, we analyze two types of data, heart rate variability (HRV), for the purpose of distinguishing healthy subjects from patients with congestive heart failure (CHF), and EEG, for the detection of epileptic seizures.

We downloaded two types of HRV data from the PhysioNet (MIT-BIH Normal Sinus Rhythm Database and BIDMC Congestive Heart Failure Database available at http://www.physionet.org/physiobank/database/#ecg), one for healthy subjects, and the other for subjects with CHF. The latter includes long-term ECG recordings from 15 subjects (11 men, aged 22 to 71, and 4 women, aged 54 to 63) with severe CHF (NYHA class 3–4). This group of subjects was part of a larger study group receiving conventional medical therapy prior to receiving the oral inotropic agent, milrinone. Further details about the larger study group can be found at the PhysioNet. The individual recordings of ECG are each about 20 h in duration, and contain two ECG signals each sampled at 250 samples per second with 12-bit resolution over a range of ±10 millivolts. The other database are for 18 normal subjects. The individual recordings are each about 25 h in duration, each sampled at 128 samples per second. The HRV data analyzed here are the R-R intervals (in unit of second) derived from the ECG recordings.

The EEG database is downloaded at http://www.meb.unibonn.de/epileptologie/science/physik/eegdata.html. The database consists of three groups, H (healthy), E (epileptic subjects during a seizure-free interval), and S (epileptic subjects during seizure); each group contains 100 data segments, whose length is 4097 data points with a sampling frequency of 173.61 Hz. These data have been carefully examined by adaptive fractal analysis (Gao et al., 2011c) and scale-dependent Lyapunov exponent (Gao et al., 2006b, 2011b, 2012), for the same purpose of distinguishing epileptic seizure EEG from normal healthy EEG.

### 2.2. Methods

Entropy characterizes creation of information in a dynamical system. To facilitate derivation of a fundamental scaling law for MSE, we first rigorously define MSE and all related concepts.

Suppose that the *F*-dimensional phase space is partitioned into boxes of size ε*^F^*. Suppose that there is an attractor in phase space and consider a transient-free trajectory $\vec{x}(t)$. The state of the system is now measured at intervals of time τ. Let *p*(*i*_1_, *i*_2_, … ,*i*_*d*_) be the joint probability that $\vec{x}(t)$ = τ) is in box *i*_1_, $\vec{x}(t)$ = 2τ) is in box *i*_2_, …, and $\vec{x}(t)$ = *d*τ) is in box *i*_*d*_. Let us now introduce the block entropy,

(1) $$H_{d}(\text{ε},\tau )=-\sum_{i_{1},\cdots ,i_{d}}p(i_{1},\cdots ,i_{d})\ln p(i_{1},\cdots ,i_{d}),$$

take the difference between *H*_*d* + 1_ (ε, τ) and *H*_*d*_ (ε, τ), and normalize it by τ,

(2) $$h_{d}(\text{ε},\;\tau )=\frac{1}{\tau }[H_{d+1}(\text{ε},\;\tau )-H_{d}(\text{ε},\;\tau )].$$

Let

(3) $$h(\text{ε},\;\tau )=\underset{d\to \infty }{\lim}h_{d}(\text{ε},\;\tau )$$

It is called the (ε, τ)-entropy (Gaspard and Wang, 1993). Taking limits, we obtain the Kolmogorov-Sinai (K-S) entropy,

(4) $$\begin{array}{l} K=\underset{\tau \to 0}{\lim}\;\underset{\text{ε}\to 0}{\lim}\;h(\text{ε},\tau )\\ \;=\underset{\tau \to 0}{\lim}\;\underset{\text{ε}\to 0}{\lim}\;\underset{d\to \infty }{\lim}\;\frac{1}{\tau }[H_{d+1}(\text{ε},\tau )-H_{d}(\text{ε},\tau )] \end{array}$$

We now consider computation of the (ε, τ)-entropy from a time series of length *N, x*_1_, *x*_2_, …, *x*_*N*_. As is well-known, the first step is to use the time delay embedding to construct vectors of the form:

(5) $$V_{i}=[x_{i},x_{i+L},...,x_{i+(m-1)L}],$$

where *m*, the embedding dimension, and *L*, the delay time, can be chosen according to certain optimization criterion (Gao et al., 2007). Then one can employ the Cohen-Procaccia algorithm (Cohen and Procaccia, 1985) to estimate the (ε, τ)-entropy. In particular, when it is evaluated at a fixed finite scale $\hat{\varepsilon }$, the resulting entropy is called the approximate entropy. To get better statistics from a finite time series, one may compute *K*_2_(ε) using the Grassberger-Procaccia’s algorithm (Grassberger and Procaccia, 1983):

(6) $$K_{2}(\text{ε})=\underset{m\to \infty }{\lim}\frac{\ln C^{\left(m\right)}(\text{ε})-\ln C^{\left(m+1\right)}(\text{ε})}{mL\delta t}$$

where δ*t* is the sampling time, *C*^(*m*)^(ε) is the correlation integral based on the *m* − dimensional reconstructed vectors *V*_*i*_ and *V*_*j*_,

(7) $$C^{\left(m\right)}(\text{ε})=\underset{N_{v}\to \infty }{\lim}\frac{2}{N_{v}(N_{v}-1)}\sum_{i=1}^{N_{v}-1}\sum_{j=i+1}^{N_{v}}H(\text{ε}-||V_{i}-V_{j}||),$$

where *N*_*v*_ = *N* − (*m* − 1)*L* is the number of reconstructed vectors, *H*(*y*) is the Heaviside function (1 if *y* ≥ 0 and 0 if *y* < 0). *C*^(*m*+1)^(ε) can be computed similarly based on the *m* + 1 − dimensional reconstructed vectors. When we evaluate *K*_2_ (ε) at a finite fixed scale $\hat{\varepsilon }$, we obtain the sample entropy *S*_*e*_ (Richman and Moorman, 2000).

MSE analysis is based on the sample entropy *S*_*e*_. The procedure is as follows. Let *X* = {*x*_*t*_: *t* = 1, 2,…} be a covariance stationary stochastic process with mean μ, variance σ^2^, and autocorrelation function *r*(*k*), *k* ≥ 0. Construct a new covariance stationary time series

$$X^{(25b_{s})}=\{x_{t}^{\left(b_{s}\right)}:t=1,2,3,\ldots \},\;\;b_{s}=1,2,3,\ldots ,$$

by averaging the original series *X* over non-overlapping blocks of size *b*_*s*_,

(8) $$x_{t}^{\left(b_{s}\right)}=(x_{tb_{s}-b_{s}+1}+\cdots +x_{tb_{s}})/b_{s},\;t\geq 1\;.$$

MSE analysis involves (i) choosing a finite scale $\hat{\varepsilon }$ in phase space, and (ii) computing *S*_*e*_ from the original and the smoothed data *X* and *X*^(*b*_*s*_)^ at the chosen scale $\hat{\varepsilon }$. For convenience of later discussion, we denote *K*_2_^(*b*_*s*_)^ (ε) for the correlation entropy of the smoothed data. When *b*_*s*_ = 1, it is the correlation entropy of the original data, and can be simply denoted as *K*_2_ (ε).

We emphasize that the length of the smoothed time series is only 1/*b*_*s*_ of the original one. To fully resolve the scaling behavior of *K*_2_ (ε), the requirement on data length is quite stringent. A fundamental question is whether MSE calculated from short noisy data is meaningful or not.

## 3. Results

### 3.1. Scaling for the MSE of fractal time series

Among the most widely used models for biological signals, including HRV, EEG, and posture (Gao et al., 2011a), is the fractal time series with long memory, the so-called 1/*f*^α^, or 1/*f*^2*H* − 1^, α = 2*H* − 1 processes, where 0 < *H* < 1 is called the Hurst parameter, whose value determines the correlation structure of the data (Gao et al., 2006a, 2007): when *H* = 1/2, the process is like the independent steps of the standard Brownian-motion; when *H* < 1/2, the process has anti-persistent correlations; when *H* > 1/2, the process has persistent correlations. Two special cases, white noise with *H* = 0.5 and 1/*f* process with *H* = 1, have been extensively used for the development of multivariate MSE (Ahmed and Mandic, 2011). In this subsection, we derive fundamental scalings for MSE of the ubiquitous 1/*f*^2*H* − 1^ noise.

A covariance stationary stochastic process *X* = {*X*_*t*_: *t* = 0, 1, 2, …}, with mean μ, variance σ^2^, and autocorrelation function *r*(*w*), *w* ≥ 0, is said to have long range correlation if *r*(*w*) is of the form Cox (1984)

(9) $$r(w)\sim w^{2H-2},\;as\;w\to \infty ,$$

where 0 < *H* < 1 is the Hurst parameter. When 1/2 < *H* < 1, ∑_*w*_ *r*(*w*) = ∞, leading to the term long range correlation. Note the *X* time series has a power spectral density 1/*f*^2*H*−1^. Its integration, {*y*_*t*_}, where *y*_*t*_ = ∑^*t*^_*i* = 1_ *x*_*i*_, is called a random walk process which is nonstationary with power-spectral density (PSD) 1/*f*^2*H*+1^. Being 1/*f* processes, they cannot be aptly modeled by Markov processes or ARIMA models (Box and Jenkins, 1976), since the PSD for those processes are distinctly different from 1/*f*. To adequately model 1/*f* processes, fractional order processes has to be used. The most popular is the fractional Brownian motion model Mandelbrot (1982), whose increment process is called the fractional Gaussian noise (fGn). The importance and popularity of fGn in modeling various types of noises in science and engineering motivates us to focus our analysis on it when deriving the bi-scaling law.

1/*f*^2*H*−1^ noises are self-similar, with the autocorrelation for the original data and the smoothed data (defined by Equation 8) being the same (Gao et al., 2006a, 2007). This signifies that there must exist a simple relation between *K*_2_^(*b*_*s*_)^ (ε) and *K*_2_ (ε). To find this relation, we note that the variance, *var*(*X*^(*b*_*s*_)^), of the smoothed data, and the variance, σ^2^, of the original data, are related by the following simple and elegant scaling law (Gao et al., 2006a, 2007),

(10) $$var(X^{\left(b_{s}\right)})=\sigma^{2}b_{s}{}^{2H-2}$$

Equation (10) states that the scale ε for the original data is transformed to a smaller scale *b*_*s*_^*H*−1^ε for the smoothed data. Using the self-similarity property of the 1/*f*^2*H*−1^ noise, we therefore obtain,

(11) $$K_{2}^{\left(b_{s}\right)}(b_{s}^{H-1}\text{ε})=K_{2}(\text{ε})$$

Since for stationary random processes, *K*_2_ (ε) diverges when ε → 0, Equation (11) states that $K_{2}^{\left(b_{s}\right)}(b_{s}^{H-1}\varepsilon )$ can be obtained from *K*_2_ (ε) by shifting downward the curve for *K*_2_ (ε). How much *K*_2_ (ε) should be shifted depends on the functional form for *K*_2_ (ε), which we shall find out momentarily.

First we note that for 1-D independent random variables, which correspond to *H* = 1/2, *h*(ε,τ) ~ − ln ε (Gaspard and Wang, 1993). Therefore, *K*_2_ (ε) ~ −ln ε. In fact, for any stationary noise process, irrespective of its correlation structure, we always have *C*^(*m*)^(ε) ~ ε ^−*m*^, ε → 0, therefore,

(12) $$K_{2}(\text{ε})\sim -\ln\text{ε},\;\;\text{ε}\to 0$$

Equation (12) is, however, not adequate for us to understand the scaling of *K*_2_ (ε) on finite scales. To gain more insights, we resort to the rate distortion function or the Shannon-Kolmogorov (SK) entropy (Berger, 1971; Gaspard and Wang, 1993). It is thought to diverge with ε in the same way as the (ε,τ)-entropy and *K*_2_ (ε) (Gaspard and Wang, 1993).

Suppose we wish to approximate the random signal *X*(*t*) by *Z*(*t*) according to

(13) $$\text{ρ}(X,Z)=\underset{T\to \infty }{\lim}\frac{1}{T}\int_{0}^{T}\langle {\left[X(t)-Z(t)\right]}^{2}\rangle dt\leq {\text{ε}}^{2}$$

where < > denotes averaging. Equation (13) may be considered a partition of the phase space containing the random signal *X*(*t*) by centering around *X*(*t*). Denote the conditional probability density for *Z* given *x* by *q*(*z*|*x*). The mutual information *I*(*q*) between *X* and *Z* is a functional of *q*(*z*|*x*),

(14) I(q)=∫∫dxdz p(x)q(z|x)ln[q(z|x)/q(z)].

The SK (ε,τ)-entropy is

(15) $$H_{SK}(\text{ε},\tau ,T)={\text{Inf}}_{q\in Q(\text{ε})}I(q)$$

where *Q*(ε) is the set of all conditional probabilities *q*(*z*|*x*) such that Condition (13) is satisfied. The SK (ε,τ)-entropy per unit time is then

(16) $$h_{SK}(\text{ε},\tau )=\underset{T\to \infty }{\lim}H_{SK}(\text{ε},\tau ,T)/T$$

For stationary Gaussian processes, *h*_*SK*_ (ε,τ) can be readily computed by the Kolmogorov formula (Berger, 1971; Kolmogorov, 1956). In the case of a discrete-time process, it reads

(17) $${\text{ε}}^{2}=\frac{1}{2\pi }\int_{-\pi }^{\pi }\min[\theta ,\Phi (\text{ω})]d\text{ω}$$

(18) $$h_{SK}(\text{ε},\tau )=\frac{1}{4\pi }\int_{-\pi }^{\pi }\max\{0,\ln[\Phi (\text{ω})/\text{θ}]\}d\text{ω}$$

where Φ(ω) is the PSD of the process and θ is an intermediate variable.

We now evaluate the SK entropy for a popular model of 1/*f*^2*H*−1^ noise, the fractional Gaussian noise (fGn). It is a stationary Gaussian process with PSD 1/ ω^2*H*−1^. Since we are primarily interested in small ε, we may choose the intermediate variable θ ≤ Φ(ω). Let us denote Φ(ω) = *B*(*H*) ω^1−2*H*^, where *B*(*H*) is a factor depending on *H*. When *H* = 1/2, it equals the variance of the noise σ ^2^_*H* = 1/2_. Using Equations (17) and (18), we immediately have

(19) $$h_{SK}(\text{ε})=A(H)-\ln\text{ε}$$

where

(20) $$A(H)=\frac{1-2H}{2}(\ln\pi -1)+\frac{1}{2}\ln B(H)$$

If we assume fGn of different *H* to have the same variance, then $\int_{0}^{\text{\textbackslash{}}\pi }\Phi (\omega )d\omega$ is a constant independent of *H*. *A*(*H*) can then be written as

(21) $$A(H)=\frac{1}{2}\ln\sigma_{H=1/2}^{2}+\frac{1}{2}[\ln(2-2H)-(1-2H)]$$

*A*(*H*) is maximal when *H* = 1/2. However, when *H* is not close to 0 or 1, the term $\frac{1}{2}$[ln (2 − 2*H*) − (1 − 2*H*)] is negligibly small, signifying that *h*_*SK*_ (ε) cannot readily classify fGn of different *H*.

Since *h*_*SK*_ (ε) and *K*_2_ (ε) diverge in the same fashion (Gaspard and Wang, 1993), using Equation (12) to determine the prefactor, we have a scaling for finite ε

(22) $$K_{2}(\text{ε})\sim -\ln\;\text{ ε}$$

Combining Equations (22) and (11), we arrive at a fundamental bi-scaling law for *K*^(*b*_*s*_)^_2_ (ε) for fractal time series:

(23) $$K_{2}^{\left(b_{s}\right)}(\text{ε})\sim (H-1)\;\ln\;b_{s}-\ln\;\text{ε}$$

**Figure 1:**
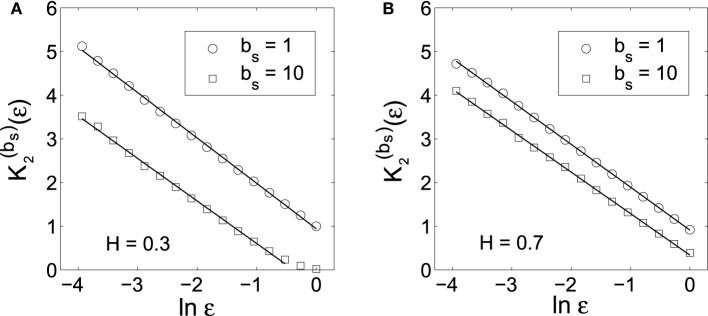
*****K***^(***b***_***s***_)^_2_ (ε) vs. ln ε curves corresponding to the original data (***b***_***s***_ = 1) and the smoothed data (***b***_***s***_ = 10) for fGn processes with (A) ***H*** = 0.3 and (B) ***H*** = 0.7**. The slopes of the linear regression lines are very close to 1.

**Figure 2:**
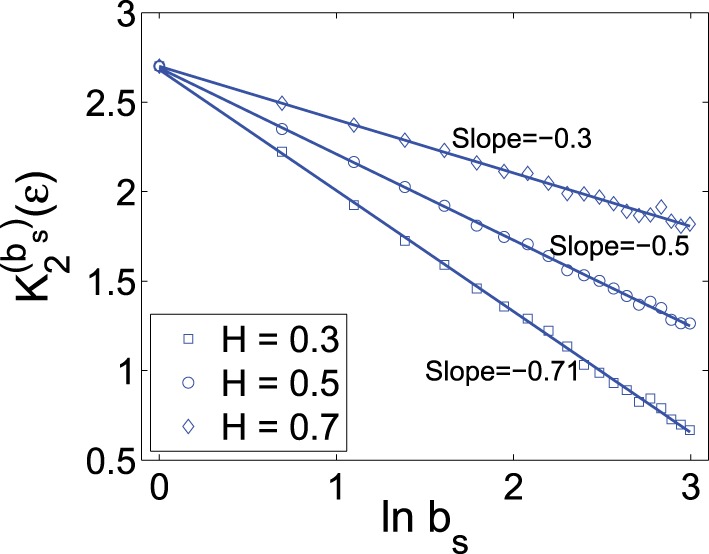
*****K***^(***b***_***s***_)^_2_ (ε) vs. ln b_***s***_ curves for fGn processes with different *H* values**. The scale ε is chosen as 20% of the standard deviation of the corresponding fGn process. *H* value is estimated as 1 plus the slope of the curve.

To verify the above bi-scaling law, and more importantly, to gain insights into the relative importance of the two scale parameters *b*_*s*_ and ε in MSE analysis, we numerically perform MSE analysis of fGn processes with different *H*. A few examples are shown in Figures 1, 2. The computations are done with 2^14^ points and *m* = 2. We observe excellent bi-scaling relations, thus verifying Equation (23). Recalling our earlier comment that *K*_2_ (ε) itself is not very useful for distinguishing fGn of different *H*, Figure 2 clearly shows that the scaling *K*^(*b*_*s*_)^_2_ (ε) ~ (*H* − 1) ln *b*_*s*_ can aptly separate fGn processes of different *H*. In fact, *H* values estimated from Figure 2 are fully consistent the values of *H* chosen in simulating the fGn processes. This analysis thus has demonstrated the major advantage of the scale parameter *b*_*s*_ over ε for the study of fGn processes using MSE. It has also made it clear that MSE is a highly non-trivial extension of the sample entropy, and more generally, the correlation entropy *K*_2_(ε).

While Equation (23) is fundamental for MSE, it can also help us better understand the behavior of multivariate MSE, which is shown in numerical simulations to be almost constant for 1/*f* processes with *H*=1, and decays in a well-defined fashion for white noise, where *H*=1/2, and some randomized data derived from experimental data possibly with correlations (Ahmed and Mandic, 2011). The reason is very clear. For 1/*f* process, *H*=1, and therefore, MSE or multivariate MSE does not vary with the scale parameter *b*_*s*_. For white noise or some derived randomized data, *H*=1/2, and therefore, MSE or multivariate MSE decays with the scale parameter *b*_*s*_ in a well-defined fashion,

(24) $$K_{2}^{\left(b_{s}\right)}(\text{ε})\sim -\frac{1}{2}\ln b_{s},\;\;\;or\;\;\;b_{s}\sim e^{-2K_{2}(\text{ε})}.$$

One can readily check that the MSE curve for white noise shown in Ahmed and Mandic (2011) is fully consistent with the formula derived here.

### 3.2. Heart rate variability data analysis

As an important application of MSE, we analyze HRV data for the purpose of distinguishing healthy subjects from patients with CHF, a life-threatening condition. This is an important issue. We refer to (Hu et al., 2009, 2010) and references therein for the background. Note that part of the data examined here were analyzed in prior work (Ivanov et al., 1999; Barbieri and Brown, 2006), for the same purpose. We analyze all 33 datasets here. For ease of comparison, we take the first 3 × 10^4^ points of both groups of HRV data for analysis. Note that based on different *b*_*s*_ parameter, MSE was not very good at separating the two groups (Hu et al., 2010). This instigated a debate on whether MSE was useful or not for analyzing HRV (Wessel et al., 2003; Nikulin and Brismar, 2004). To resolve this interesting debate, and more importantly, to satisfactorily separate the two groups of HRV data, we shall focus on the dependence of MSE on the scale parameter ε in the following discussions.

**Figure 3:**
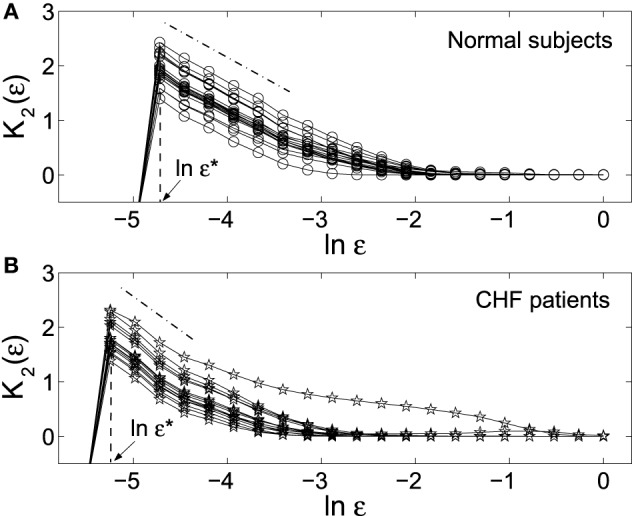
*****K***_2_ (ε) vs. ln ε curves for the HRV data of (A) 18 normal subjects and (B) 15 patients with CHF**. Each curve corresponds to one subject. The computations were done with 3 × 10^4^ points and *m* = 5. ε^*^ indicates the smallest scale resolvable by the data.

Since earlier studies find HRV data to be nonstationary, having 1/*f* spectrum with anti-persistent long-range correlations and multifractality (see Ivanov et al., 1999 and references therein), we analyze the increment processes of the HRV data. Figure 3 shows *K*_2_ (ε) vs. ln ε curves for the two groups of HRV data. We observe: (i) On small scales, *K*_2_ (ε) vs. ln ε curves for both groups of HRV data show good scaling behavior. As a consequence, one can expect a scaling relation between *K*^(*b*_*s*_)^_2_ (ε) and ln *b*_*s*_ (Equation 23). This is indeed so. The results, being very similar to that shown in Figure 2, are not shown here, however. (ii) The scaling of *K*_2_ (ε) vs. ln ε is better and longer for the normal HRV data. (iii) As indicated by ε^*^ in the figure, the smallest scale resolvable by the HRV data of the healthy subjects is much larger than that of the diseased subjects.

We now discuss how to use MSE to distinguish the healthy subjects from patients with CHF. We have found (i) The curves *K*^(*b*_*s*_)^_2_ (ε) vs. *b*_*s*_ averaged over all the subjects within the two groups are different, just as reported in Costa et al. (2005). However, such curves are not very useful for separating the two groups as a diagnostic tool, as pointed out in Nikulin and Brismar (2004). The fundamental reason is of course that the Hurst parameter *H* is not very effective in distinguishing healthy subjects from patients with HRV, as quantitatively analyzed in Hu et al. (2010). (ii) The smallest resolvable scale, ε^*^, completely separates the healthy subjects from patients with CHF, as shown by Figure 3. Note the scale parameter ε is a generalization of the concept variance (or standard deviation). The observation made by Nikulin and Brismar (2004) that a variance-like parameter is better than MSE with varying block size parameter *b*_*s*_ in distinguishing healthy subjects from patients with HRV is most appropriately interpreted as the following: the parameter *b*_*s*_ is less important than the scale parameter ε. This is somewhat the opposite of the case for 1/*f* noise analyzed in the last section.

**Figure 4:**
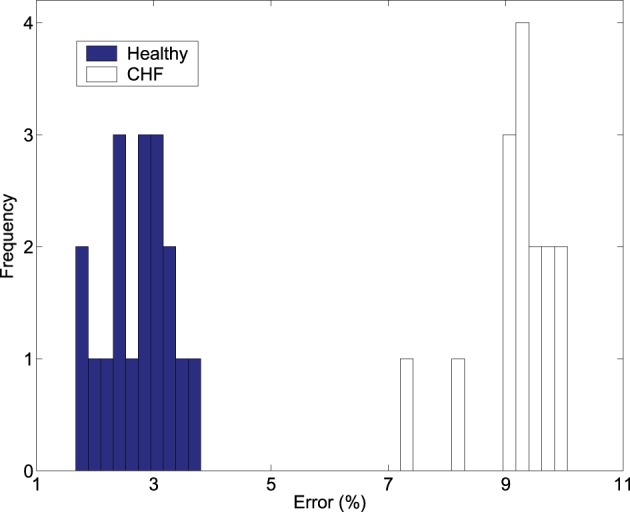
The frequency of the percentage of errors obtained by linearly fitting the ***K***_2_ (ε) vs. ln ε curves in Figure 3 with 6 points starting from ε ^*^ for the healthy and diseased subjects.

To more clearly see how much more advantageous ε is over *b*_*s*_ in distinguishing healthy subjects from patients with HRV, we examine how the scaling *K*_2_(ε) ~ − ln ε can be used for this purpose. We have found that the errors obtained by linearly fitting the *K*_2_(ε) vs. ln ε curves of Figure 3 are much smaller for the normal HRV data than for those of CHF patients and also can completely separate the healthy subjects from patients with CHF. This is shown in Figure 4. Therefore, the scale parameter ε is indeed more important than *b*_*s*_.

### 3.3. Epileptic seizure detection through MSE of EEG

Epilepsy is a common and debilitating brain disorder. It is characterized by intermittent seizures. During a seizure, the normal activity of the central nervous system is disrupted. The concrete symptoms include abnormal running/bouncing fits, clonus of face and forelimbs, or tonic rearing movement as well as simultaneous occurrence of transient EEG signals such as spikes, spike and slow wave complexes or rhythmic slow wave bursts. Clinical effects may include motor, sensory, affective, cognitive, automatic and physical symptomatology. To make medications effective, timely detection of seizure is very important. In the past several decades, considerable efforts have been made to detect/predict seizures through nonlinear analysis of EEGs. For a list of the major nonlinear methods proposed for seizure detection, we refer to Gao and Hu (2013) and references therein. In particular, the three groups of EEG data analyzed here, H (healthy), E (epileptic subjects during a seizure-free interval), and S (epileptic subjects during seizure), were examined by adaptive fractal analysis (Gao et al., 2011c) and scale-dependent Lyapunov exponent (Gao et al., 2012), and excellent classification was achieved.

**Figure 5:**
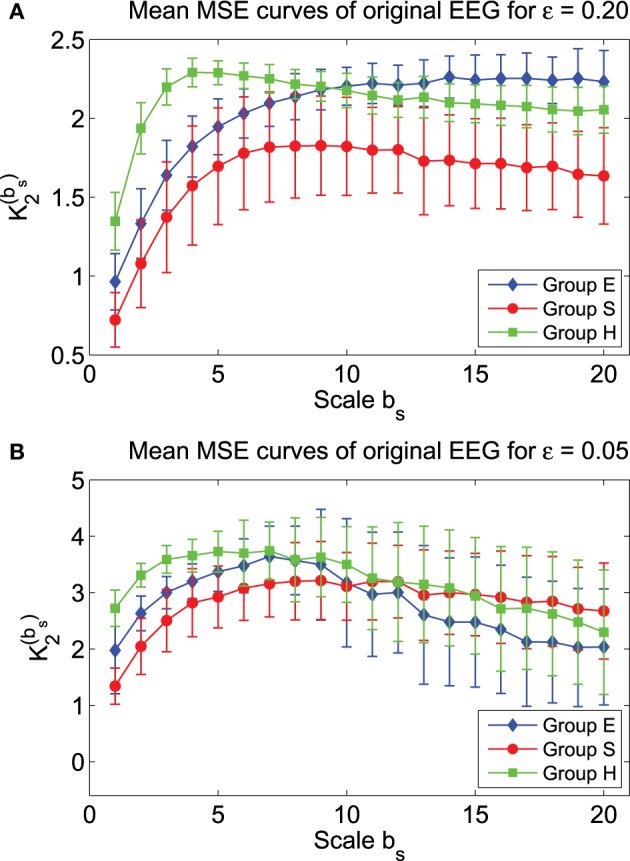
**Mean MSE curves for the 3 EEG groups with (A) ε = 0.2 and (B) ε = 0.05**.

To examine how well MSE characterizes the three groups of EEG data, we have plotted in Figure 5 the mean MSE curves for the three groups, for two parameter values of the phase space scale, ε. We observe that they separate very well. Indeed, statistical test shows that the separations are significant. In particular, for the scale parameter in the phase space ε = 0.2, the MSE curve for the S group lies well below the other 2 curves. One may be tempted to equate this as smaller complexity of the seizure EEG. However, such an interpretation is informative only relative to the specific ε chosen here, which is 0.2. When ε = 0.05, the red curve for seizure EEG actually lie above the other 2 curves for larger *b*_*s*_. In fact, if one can pause a moment and think twice, one would realize that such interpretations are not too helpful for clinical applications, since MSE can vary substantially within and across the groups.

**Figure 6:**
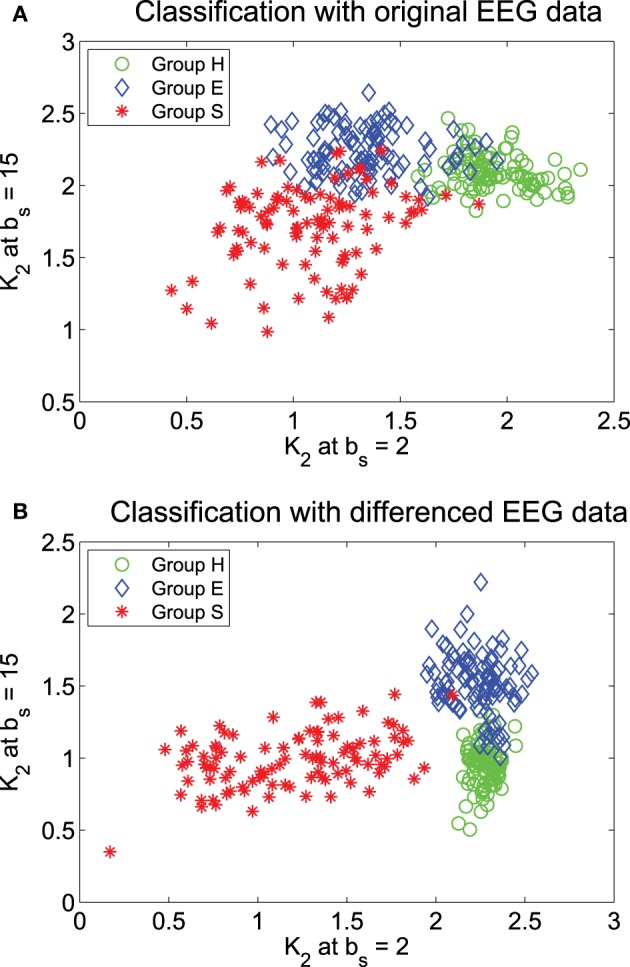
**Classification of the 3 EEG groups using features from the MSE curves: (A) the original data and (B) the differenced data**.

We have tried to use MSE at specific *b*_*s*_ values to classify the three groups of EEG. Guided by the mean MSE curves in Figure 5, we have found that when ε = 0.2, if only two *b*_*s*_ can be used, then *b*_2_ = 2 and 15 are the optimal values. The result of the classification is shown in Figure 6A. We observe that there are some overlaps between groups H (healthy) and E (epileptic subjects during a seizure-free interval), as well as E and S (epileptic subjects during seizure). Intuitively, this is reasonable. Overall, the classification is not very satisfactory. How may we improve the accuracy of the classification?

**Figure 7:**
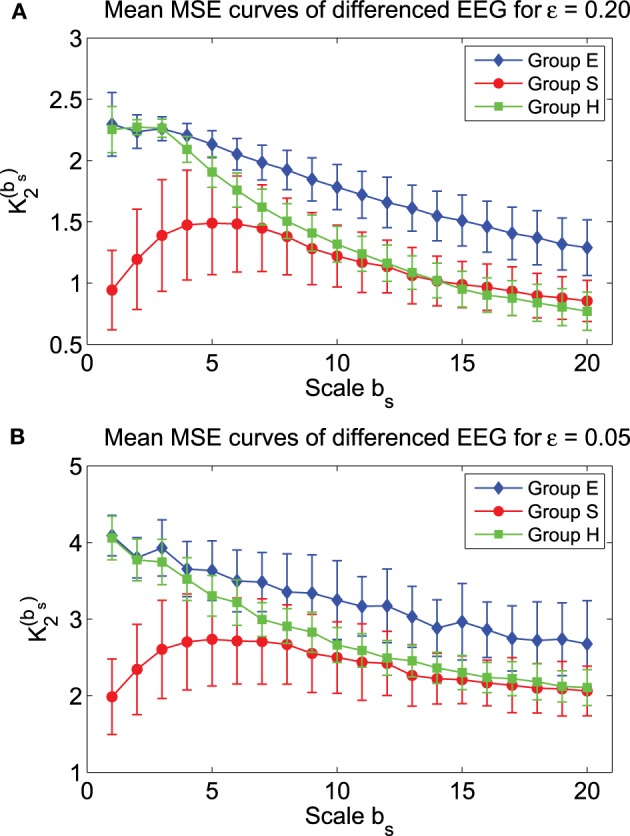
**Mean MSE curves for the differenced data of the 3 EEG groups with (A) ε = 0.2 and (B) ε = 0.05**.

Recall that in fractal scaling analysis of EEG, EEG data are found to be equivalent to random walk processes, but not noise or increment processes (Gao et al., 2011c). The latter amounts to a differentiation of the random walk processes. Since the basic scaling law derived here is for noise or increment process, but not for random walk processes, it suggests us to try to compute MSE from the differenced data of EEG, defined by *y*_*i*_ = *x*_*i*_ − *x*_*i* − 1_, where *x*_*i*_ is the original EEG signal. The mean MSE curves for the differenced data of EEG are shown in Figure 7, again for two ε values. We observe that the separation between the mean MSE curves becomes wider. Indeed, classification of the 3 EEG groups now is much improved, as shown in Figure 6B. It should be noted however that the accuracy of the classification is still slightly worse than using other methods, such as adaptive fractal analysis (Gao et al., 2011c) and scale-dependent Lyapunov exponent (Gao et al., 2012).

## 4. Conclusion and discussion

To better understand MSE, we have derived a fundamental bi-scaling relation for the MSE analysis. While MSE analysis normally only focuses on the scale parameter *b*_*s*_ with ε more or less arbitrarily chosen, our analysis of fGn and HRV data clearly demonstrates that both scale parameters are important—in the case of HRV analysis, the ε is more important, while in the case of 1/*f* noise, the *b*_*s*_ parameter is more important. In fact, we have shown (Hu et al., 2010) that MSE, when used with ε fixed, is not very effective in distinguishing healthy subjects from patients with HRV. The accuracy achieved when we focus on the scaling of *K*_2_(ε) ~ −ln ε is not only much higher, but also comparable to that using the scale-dependent Lyapunov exponent (SDLE) (Gao et al., 2006a, 2007, 2013), as reported by Hu et al. (Hu et al., 2010). The fundamental reason of course is that SDLE has a similar scaling as *K*_2_(ε) ~ −ln ε.

We have also computed MSE for the original as well as the differenced data of the three EEG groups, H (healthy), E (epileptic subjects during a seizure-free interval), and S (epileptic subjects during seizure), and found that mean MSE curves for the three groups are well separated. The classification of the 3 EEG groups using MSE at two specific scale parameters *b*_*s*_ is reasonably good, and is better for the differenced data than for the original EEG data. This strongly suggests that EEG data are like random walk processes. However, even with the differenced data of EEG, the classification is still not as accurate as using adaptive fractal analysis (Gao et al., 2011c) and scale-dependent Lyapunov exponent (Gao et al., 2011a). One of the reasons for this inferiority lies in the difference in the range of scales covered by these three multiscale methods. Adaptive fractal analysis and scale-dependent Lyapunov exponent both cover the entire range of scales presented in the EEG data. However, with the length of the EEG data, which is only 4097 points for each data set, MSE can only cover a moderate range of scales, with the largest *b*_*s*_ only around 20, since with *b*_*s*_ = 20, the smoothed data is already only 200 points long. Our analysis here has raised an important question: how do we use MSE to analyze short data? We conjecture that it may be beneficial to focus on the scaling of *K*_2_(ε) ~ −ln ε, or develop new smoothing schemes, by introducing a parameter equivalent to 1/*b*_*s*_ but without sacrificing the length of the smoothed data.

### Conflict of interest statement

The authors declare that the research was conducted in the absence of any commercial or financial relationships that could be construed as a potential conflict of interest.
